# Equilibrium of Tiered Healthcare Resources during the COVID-19 Pandemic in China: A Case Study of Taiyuan, Shanxi Province

**DOI:** 10.3390/ijerph19127035

**Published:** 2022-06-08

**Authors:** Xueling Wu, Ruiqi Mao, Xiaojia Guo

**Affiliations:** School of Geographical Sciences, Shanxi Normal University, Taiyuan 030031, China; wuxueling@sxnu.edu.cn (X.W.); 220113033@sxnu.edu.cn (R.M.)

**Keywords:** medical and health resources, tiered healthcare system, spatial equilibrium, POI data, Geodetector

## Abstract

COVID-19 has caused more than 500 million infections and 6 million deaths. Due to a continuous shortage of medical resources, COVID-19 has raised alarm about medical and health resource allocation in China. A balanced spatial distribution of medical and health resources is a key livelihood issue in promoting the equalization of health services. This paper explores the spatial allocation equilibrium of two-tier medical and health resources and its influencing factors in Taiyuan. Using extracted POIs of medical and health resources of AMAP, we evaluated the spatial quantitative characteristics through the Health Resources Density Index, researched the spatial distribution pattern by kernel density analysis, hot spot analysis, and service area analysis, and identified the influencing factors of the spatial distribution equilibrium by the Geodetector model. The findings are as follows. The overall allocation level of medical and health resources in Taiyuan is low. There are tiered and regional differences; the response degree of primary care facilities to external factors is greater than that of hospitals; and the comprehensive influence of economic and topographic systems is crucial compared with other factors. Therefore, in order to promote the rational spatial distribution of medical and health resources in Taiyuan and to improve the construction of basic medical services within a 15 min radius, it is important to continuously improve the tiered healthcare system, uniformly deploy municipal medical and health resources, and increase the resource allocation to surrounding counties and remote mountainous areas. Future research should focus on collecting complete data, refining the research scale, analyzing qualitative differences, and proposing more accurate resource allocation strategies.

## 1. Introduction

China has eliminated poverty, and people’s physical needs are basically met. According to Maslow’s hierarchy of needs, people’s safety needs, namely, people’s health, has become important. However, as urbanization accelerates, the imbalance between the growing demand for multi-level diversified medical and health services and the limited supply of medical and health resources has become more prominent [1]. The division and cooperation mechanism among medical and health institutions is not wholesome and lacks connectivity and sharing. There is insufficient cooperation and synergy among all tiers and all types of healthcare institutions. It is difficult for the service system to effectively respond to the increasing incidence of chronic and high morbidity diseases, especially sudden public infectious diseases, which have a substantial impact on the medical system and occupy the diagnosis and treatment resources needed for other chronic diseases [2].

Since 2020, COVID-19 has affected 215 countries around the world, with more than 6 million dead cases, and has posed substantial threats to healthy people globally. With China’s large population base and frequent mobility, the concealment of virus transmission has caused large-scale diffusion in 31 provinces nationwide, which has impacted health and social stability in China [3]. During the COVID-19 pandemic, the tiered healthcare system plays an important role in ensuring the original treatment of chronic diseases while responding to the large number of confirmed patients and asymptomatically infected patients [4]. Its implementation is conducive to ensuring that different tiers of medical and health resources undertake the treatment of different diseases, strengthening regional resource integration, avoiding resource squeezes, and maximizing resource benefits.

China has issued the Healthy China Strategy in the Report of the 19th CPC National Congress, and a series of development plans for the health service system by the State Council illustrates the increasing attention at the national level to the livelihood issue of basic medical and health services [5]. However, medical and health resources are inadequately invested and unevenly allocated in China. It is a serious challenge for China to consolidate the victory against the epidemic and to universalize the Healthy China Strategy benefits. This requires regions to pay attention to the equilibrium allocation of medical and health resources [6]. The equilibrium spatial allocation of two-tier medical and health resources is important because (1) it is conducive to improving the fairness and efficiency of resource allocation, and (2) it is conducive to ensuring the coverage of resources meets the medical needs of residents with different diseases.

The right to health is one of the basic tasks of modern countries and must be guaranteed. The right to health includes the requirement to ensure access to medical treatment and to guarantee healthcare for the population. Medical resources provide medical services, focusing on disease treatment [7], while healthcare resources provide healthcare services, focusing on disease prevention [8]. In China, medical and healthcare resources of all types mostly undertake the function of both disease treatment and disease prevention [9]. In the actual research process, medical resources and healthcare resources are not strictly distinguished [10]. Therefore, the medical and health resources mentioned in this paper refer to both medical resources and healthcare resources. During the COVID-19 pandemic, various medical and health resources not only provide treatment services, such as routine treatment and COVID-19 treatment, but also provide healthcare services, such as routine healthcare, nucleic acid detection, and vaccination. Healthcare is one of the basic tasks of modern countries. Scholars in Japan, Poland, Austria, and Brazil have investigated and analyzed the allocation of healthcare resources in their countries, and it is proposed that the quantity of healthcare resources is balanced and that healthcare resource utilization is optimized [11,12,13,14].

The unequal spatial distribution of medical and health resources is widespread in various countries, which is mainly manifested in the prominent imbalance between supply and demand [15,16] and with significant regional characteristics [17]. Especially in rural areas [18], remote areas [19], and other areas with a low level of socio-economic development [20,21], resource allocation is seriously inadequate, so it is difficult and expensive to see a doctor. Coupled with the uneven distribution of medical and health resources, China has proposed a tiered healthcare system, which has promoted the good, tiered flow of medical and health resources to a certain extent. However, the primary care facilities still have a weak service capacity, and large hospitals are overcrowded. Chinese scholars have mainly conducted research on the spatial–temporal variation characteristics of resource allocation based on urban–rural and regional differences. There is a polarization in the allocation of rural and urban medical and health resources in China, with a lack of resources and insufficient health investments in rural areas [1,7]. The regional allocation of resources is poorly balanced, with a large number of high-quality medical resources concentrated in large cities and central areas [22,23,24]. In recent years, some scholars have focused on the tiered differences in resource allocation, arguing that the polarization of medical resources between primary care facilities and hospitals is prominent and that medical resources are allocated in an inverted triangle among multi-tier healthcare institutions [5,25,26]. However, most of them are based on a national scale and lack an in-depth analysis of tiered differences at the small regional scale.

Political, economic, social, and other regional conditions will impact medical and health resource allocation. Exploring the factors of resource allocation is conducive to improving regional public service equalization. Therefore, scholars have specifically analyzed the influencing factors of the spatial distribution equilibrium of resources, the main methods including the fixed effect model [1], the gray correlation model [22], the Geodetector model [25], the spatial regression model [27], and the state space model [28]. The quantitative analysis results confirm that the level of economic development is the most important factor. In addition, the social environment, demographic characteristics, government policies, differences in residents’ conditions, and health levels also have effects on resource allocation [25,27,28,29,30,31]. However, in the process of influencing factor analysis, there is a lack of comprehensive index system construction, and the mechanism of each factor has not been deeply explored.

Point of interest (POI) data have the advantages of being real time, high precision, with wide coverage and easy access. Based on this, a series of spatial analyses can be carried out, such as kernel density analysis, cluster analysis, buffer analysis, standard deviation ellipse analysis, location entropy analysis, and hot spot analysis, which can be applied to spatial pattern research, central hot spot identification, and urban functional planning [32,33,34,35,36,37,38,39]. Therefore, a series of spatial analyses can be carried out using the POIs of medical and health resources to evaluate the spatial allocation of medical and health resources in Taiyuan. Moreover, the research on the influencing factors of spatial pattern differences based on POI data mainly adopts qualitative analysis, which lacks a quantitative analysis of regional economic and social indicators and realistic spatial distribution.

## 2. Overview of the Study Area

Taiyuan is located in the center of the Shanxi Province and the north of Taiyuan Basin, with the geographical coordinates of 37°27′~38°25′ N and 111°30′~113°09′ E. It is surrounded by mountains on the east, west, and north sides, and the center and southern areas consist of Fenhe River valley plains. With larger relief, the terrain is high in the north and low in the south and is predominantly mountainous and hilly. As the capital of Shanxi, Taiyuan is the political, economic, cultural, and medical center. It covers six districts (Jiancaoping, Xinghualing, Wanbailin, Yingze, Xiaodian, and Jinyuan), three counties (Qingxu, Yangqu, and Loufan) and Gujiao city. Taiyuan covers a total area of 6988 square kilometers and has a permanent population of 5,304,061. In 2020, the GDP of Taiyuan was CNY 415.325 billion, and per capita GDP was CNY 90,698. The population density of Taiyuan was 549 people per square kilometer, but there were significant differences among districts and counties. The population density of Yingze, Xinghualing, and Xiaodian was high, with 4681, 3644, and 2353 people per square kilometer, respectively, while that of Gujiao, Loufan, and Yangqu was low, with 136, 98, and 74 people per square kilometer, respectively.

In 2019, Taiyuan had 10.4 health technicians and 7.8 medical beds per thousand people, which were 1.43 and 2.43 times the national average, respectively. There were 3898 health institutions in the city, including 158 hospitals, 3689 primary care institutions, 40 public health professional institutes, and 11 other health institutions. Because of the primacy distribution of the urban scale, Shanxi shows strong resource agglomeration. In particular, as it is the capital city of Shanxi, medical and health resources in Taiyuan are distributed in the central urban area, resulting in an uneven resource distribution. In addition, Taiyuan is a typical mountainous city, with the main urban area located in the basin and valley area and the surrounding areas mostly mountainous, which is in line with the site selection elements and topographical characteristics of many cities in China. The city has reference significance with respect to other mountainous cities for the study of resource layout characteristics, so Taiyuan was chosen as the study area.

## 3. Data Source and Methodology

### 3.1. Data Source

**Figure 1:**
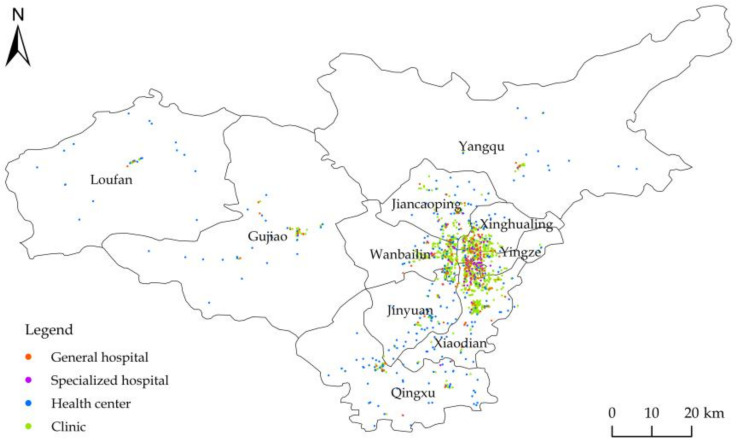
Spatial distribution of medical and health resources in Taiyuan.

Taking the districts and counties as basic spatial units, this paper analyzes the spatial distribution characteristics of two-tier medical and health resources in Taiyuan. We extracted POIs of medical and health resources based on the healthcare service category in the POI classification of AMAP on 16 April 2021. A total of 2078 items of data were extracted in 16 sub-categories, i.e., attributes, including name, region, latitude and longitude, type, and other information. A total of 1879 POI data were obtained after eliminating duplicate data and irrelevant data. According to the Industrial Classification for National Economic Activities (GB/T 4754-2017) and AMAP POI classification, combined with the service scope and department settings of various medical institutions, the POI data of medical and health resources were divided into two tiers and four types (Table 1), and their spatial distribution is shown in Figure 1. Due to the large spatial differences in population distribution and the administrative area of Taiyuan, we evaluated the level of medical and health resources in each area through the per capita index and the area mean index. The Taiyuan administrative boundary map was extracted from the National Catalogue Service for the Geographic Information System (https://www.webmap.cn/main.do?method=index, accessed on 19 November 2021) at 1:1,000,000, from the National Basic Geographic Database (2017), on 19 November 2021. The traffic data, including road information about expressways, national highways, provincial highways, county highways, township roads, and urban roads in Taiyuan, came from AMAP. The relief degree of the land surface data came from the Relief Degree of Land Surface Dataset of China (1 km) [40], and other socio-economic statistics were taken from the Taiyuan Statistical Yearbook in 2020.

### 3.2. Index System

In order to analyze the influencing factors of the spatial distribution equilibrium of the two-tier medical and health resources in Taiyuan, we took the Health Resources Density Index (HRDI) as the dependent variable and constructed an index system from five systems: the economy, the population, finance, medical insurance, and the terrain (Table 2).

### 3.3. Research Methods

The HRDI was calculated to reflect the allocation of medical and health resources in Taiyuan according to population and area. GIS spatial analysis was used to study the spatial distribution characteristics of resources, and the Geodetector model was used to analyze the influencing factors of the equilibrium.

Comprehensively, considering the influence of population and area, the HRDI reflects the allocation of medical and health resources in Taiyuan. The per capita index, area mean index, and HRDI of two-tier medical and health resources in districts and counties in Taiyuan were calculated, so as to judge the inter-county differences in the spatial distribution of medical and health resources in Taiyuan. It was calculated by the following formula [41]:

(1) $$\mathrm{HRDI}=\sqrt{\frac{\mathrm{total}\;\mathrm{health}\;\mathrm{resources}}{\mathrm{popolation}}\times \frac{\mathrm{total}\;\mathrm{health}\;\mathrm{resources}}{\mathrm{area}}}$$

Kernel density analysis embodies the overall spatial distribution and agglomeration of medical and health resources. The method centers on each sample point, *i* (x, y), and calculates its density contribution to the midpoint of each grid within the specified radius by the kernel function. The closer the distance, the greater and more intensive the density contribution [35]. Based on the POI data of the two-tier and four-type medical and health resources in Taiyuan, taking a search radius of 2.5 km, ArcGIS (Environmental Systems Research Institute, Inc., RedLands, CA, USA) was used to divide them into five grades according to natural breaks (Jenks) to obtain a kernel density map of medical and health resources in Taiyuan.

Hot spot analysis (GetisOrd Gi*) is a local spatial autocorrelation index based on the distance weight matrix, which aims to identify regions with statistically significant clustering. It works by calculating the local sum of an element and its adjacent elements within a given distance and comparing it with the sum of all elements to find high and low aggregation areas, i.e., the hot spot and the cold spot. The analysis reflects the significance level through a *p*-value confidence test and reflects the agglomeration by the Z-value. A high aggregation area has a positive and significant Z-value, while a low aggregation area has a negative and significant Z-value. The higher the absolute value of the Z-value, the closer the cluster [35,38]. In this study, orange indicates hot spots, blue indicates cold spots, and the color depth indicates their degree. *p*-values passing the 95% and 90% confidence tests are indicated by the core region and the peripheral region, respectively. *p*-values failing the 90% confidence test are shown in the middle region (white).

Service area analysis is mostly used to study the service scope of public facilities in urban planning. The coverage scope of medical and health resources can be expressed by the time required to travel from any point in a region to the nearest medical institution. The different levels of road speed were assigned, and a Taiyuan road network dataset was constructed. Network analysis in ArcGIS was used to derive the service areas formed by all streets accessible within 15 and 30 min from each medical and health resource point. A service area map of two-tier and four-type medical and health resources in Taiyuan was drawn, and the service area sizes and their ratios of all tiers and types of medical and health resources were counted to evaluate the construction of medical services in a 15 min radius. Travel speed was determined according to the Technical Standard of Highway Engineering (JTG B01-2014), a code for the design of urban road engineering (CJJ37-2012), and the relevant facts pertaining to Taiyuan (Table 3).

Geodetector (Microsoft, Redmond, Washington, DC, USA) is a set of statistical methods that detect spatial differentiation and reveal its driving forces, which can effectively detect the causes and mechanisms of spatial patterns of geographical elements [42,43,44]. We used the factor detection method of Geodetector to study the influencing factors of the spatial distribution equilibrium of two-tier medical and health resources in Taiyuan. Seventeen detection factors were selected from five systems: the economy, the population, finance, medical insurance, and the terrain, and the HRDI was taken as the dependent variable. The detection factors were discretized by the natural breaks (Jenks) method and were divided into five categories based on the maximum difference. Based on ArcGIS 10.8, 27,524 effective sampling points were extracted with an interval of 500 × 500 m. The Geodetector model was used to calculate the influence of each detection factor on the spatial distribution of the primary care facilities and hospitals, with the following formula [45]:

(2) $$q=1-\frac{\sum_{h=1}^{L}N_{h}\sigma_{h}^{2}}{N\sigma^{2}}=1-\frac{\mathrm{SSW}}{\mathrm{SST}}$$

where *q* indicates the influence of a detection factor on the spatial distribution equilibrium of medical and health resources; *N_h_* and *N* are the sample sizes of factor *h* and Taiyuan, respectively; *h* = 1, 2, ..., *L*; *σ_h_* and *σ* are the variances of the HRDI of class *h* and Taiyuan, respectively; SSW and SST are the sum of the variance within the class and the total variance of Taiyuan, respectively. The value range of *q* is from 0 to 1. The higher the *q*, the greater the influence of this factor on the spatial distribution equilibrium of medical and health resources.

Interviews were conducted from 25 to 26 May 2022, and the subjects included the current state of medical and health resources and services in Fenglingdi Village, Loufan, Taiyuan, controlling the impact of COVID-19 on medical treatment, preferences for medical treatment behavior, the accessibility of health and health resources, and reasons for difficult medical treatments. A total of 23 villagers from Fenglingdi were selected for the interviews, including 15 males and 8 females, with an age range of 19–59. Fenglingdi is situated at the foot of the Fengling Mountains, far from the urban area of Taiyuan and Loufan, and lacks medical and health resources. Mountain roads are difficult to access. The village can be considered a typical small area.

## 4. Results

### 4.1. Spatial Quantity Characteristics

As shown in Table 4, the spatial quantity of medical and health resources in Taiyuan presented the following characteristics:

(1) The allocation of medical and health resources in urban areas was superior to that of the surrounding counties. The number of medical institutions and their per capita index, area mean index, and HRDI on the whole, including primary care facilities and hospitals in the six urban districts of Taiyuan, were higher than those in the surrounding counties. The per capita index, area mean index, and HRDI of Qingxu, Gujiao, Yangqu, and Loufan were lower than the average level of Taiyuan. Among them, Xiaodian and Yingze had a rich configuration, while Yangqu and Loufan were obviously short on resources.
(2) The spatial disequilibrium of the area mean allocation was more pronounced than that of per capita allocation. The per capita allocation of primary care facilities was highest in Xiaodian at 0.735 and lowest in Yangqu at 0.222, with a difference of 3.3-fold. The regions with the highest and lowest area mean allocation were Xiaodian (1.729) and Yangqu (0.017), respectively, with a substantial difference of 104.7-fold. The per capita hospital allocation was highest in Xiaodian (0.115), 4.8 times that of the lowest in Loufan (0.024). The area mean index of hospitals in Yingze was the highest at 0.453, 192.7 times higher than that of Loufan. This means that medical and health resources, be they primary care facilities or hospitals, configured according to a different weight assignment of the population and geographic area, would directly affect allocation decisions.
(3) The HRDI at the primary care facilities was significantly higher than that at the hospitals, and the area mean index had more influence on the relative dominance of the HRDI. The primary care facilities were far higher in number than the hospitals, so the HRDIs of the primary care facilities in all regions were significantly higher than those of the hospitals, especially in Xiaodian, where the density index difference between the tiers was the greatest (0.950). The ranking of HRDIs at the primary care facilities and hospitals in each district and county was not completely consistent. Xiaodian had the highest primary care facility allocation, with the highest HRDI at 1.127, while Yingze had the most hospitals, with the highest HRDI of 0.177. The ranking of HRDIs in all the districts and counties was consistent with that of area mean indices, except with respect to the primary care facilities in Wanbailin and Xinghualing.

### 4.2. Spatial Distribution Characteristics

#### 4.2.1. Kernel Density Distribution

**Figure 2:**
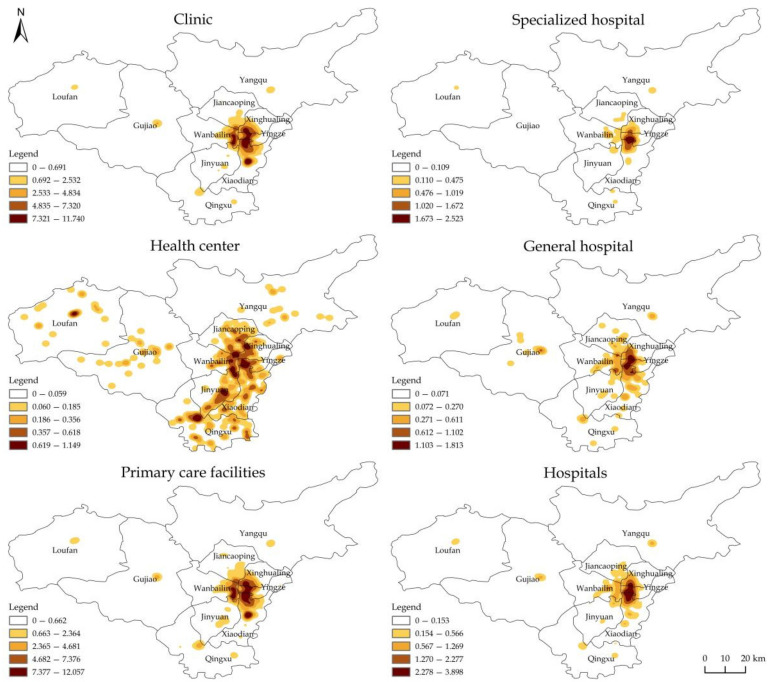
The kernel density map of two-tier and four-type medical and health resources in Taiyuan.

The spatial distribution characteristics of the kernel density of two-tier and four-type medical and health resources in Taiyuan are as follows (Figure 2). Overall, the agglomeration areas of medical and health resources at all tiers and types covered the southwest of Yingze and the northwest of Xiaodian.

The clinics were mainly concentrated in the mid-eastern part of Taiyuan. The rhombus agglomeration area involved the southeast of Jiancaoping, the southwest of Xinghualing, the west of Yingze, the northwest of Xiaodian, and the east of Wanbailin. In central Loufan, central Gujiao, central-southern Yangqu, central-eastern and northwestern Qingxu, and central-eastern Jinyuan, resources were point clustered in isolated islands. There were several hot spots of health centers in Taiyuan, among which the highest-density areas were located in the middle of Loufan, the southeast of Jiancaoping, the mid-west of Xinghualing, the west of Yingze, the southeast of Wanbailin, the mid-east of Jinyuan, and the mid-north of Qingxu. The spatial distribution pattern of the kernel density of primary care facilities was closer to that of clinics, but the agglomeration size was larger, and there were sporadic cluster areas in the southern part of Xiaodian and the eastern and western parts of Qingxu.

Specialized hospitals formed a banded agglomeration area in Xinghualing, Yingze, western Xiaodian, and eastern Wanbailin. In addition, there were several small agglomeration points in central Loufan, central and southern Yangqu, southeastern Jiancaoping, northeastern Jinyuan, and central and northern Qingxu. Compared with specialized hospitals, general hospitals had a larger agglomeration area and more agglomeration centers, forming a patchy concentrated contiguous agglomeration area in the central urban area. The hospitals were mainly concentrated in the central urban area (east to the East Intermediate Ring, Jingyuan Road and Songzhuang Road, west to the West Intermediate Ring, south to the Longcheng Street, and north to the North Intermediate Ring) and extended northward to Chaihua Line, Jinqiao Street, and Xincheng South Street in Jiancaoping. Due to the influence of the eastern area and the administrative division management, the gradient decline speed of the agglomeration area in the east was higher than that in other directions, and there were one or two point agglomeration areas in each surrounding county.

#### 4.2.2. The Hot Spot and the Cold Spot

**Figure 3:**
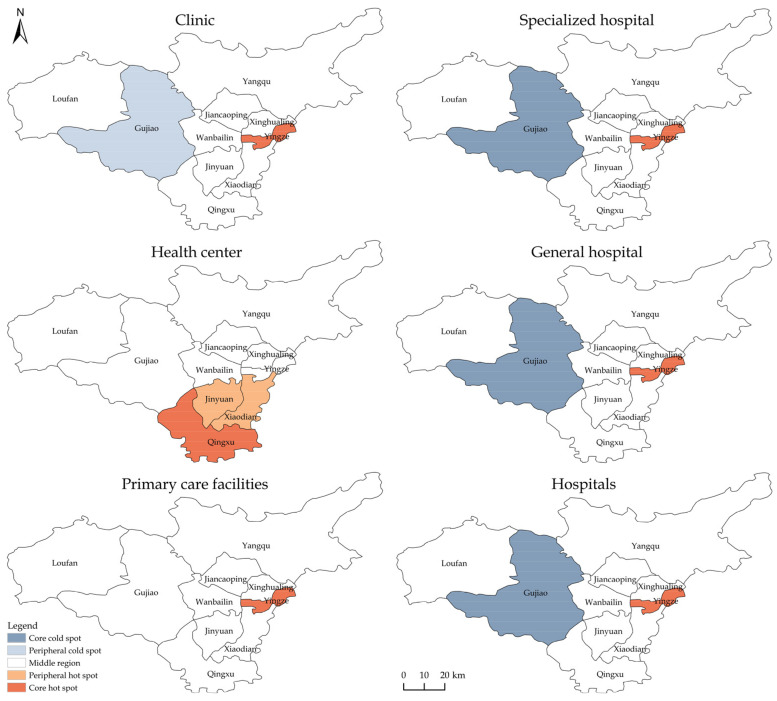
Hot spots and cold spots of two-tier and four-type medical and health resources in Taiyuan.

Figure 3 depicts the hot spots and cold spots of the two-tier and four-type medical and health resources in Taiyuan. It can be seen that the core and peripheral regions of the two-tier and four-type medical and health resources might be missing. The *p*-values of each district and county failed the 99% confidence test, indicating that the resource agglomeration of Taiyuan was generally not high. The hot spots mostly appeared in Yingze, indicating that Yingze had relatively high resource agglomeration.

Specifically, the hot spot of the clinics was Yingze, and the cold spot appeared in Gujiao, but the cold spot degree was relatively low, and the Z-value was only −1.22. There was no cold spot of health centers, and the hot spots appeared in Xiaodian, Jinyuan, and Qingxu, with Z-values of 1.69, 1.88, and 1.97, respectively, indicating that they were the main high agglomeration areas of health centers. The hot spot degree of Qingxu was the highest. The hot spot of primary care facilities was in Yingze, with a Z-value of 2.30, while the cold spot was not significant, indicating that primary care facilities were concentrated in Yingze with a high agglomeration degree.

The hot spots of specialized hospitals, general hospitals, and hospitals generally appeared in Yingze, with Z-values of 2.51, 2.39, and 2.51, respectively. All cold spots were in Gujiao, and the Z-values were −2.02, −2.12, and −2.12, respectively, without significant differences in the agglomeration degree. The regions with a high agglomeration of hospitals were mainly in Yingze and those with a low one were mainly in Gujiao. The other districts and counties were in the middle region, with a more uniform resource allocation and no obvious agglomeration.

#### 4.2.3. The Service Area

**Figure 4:**
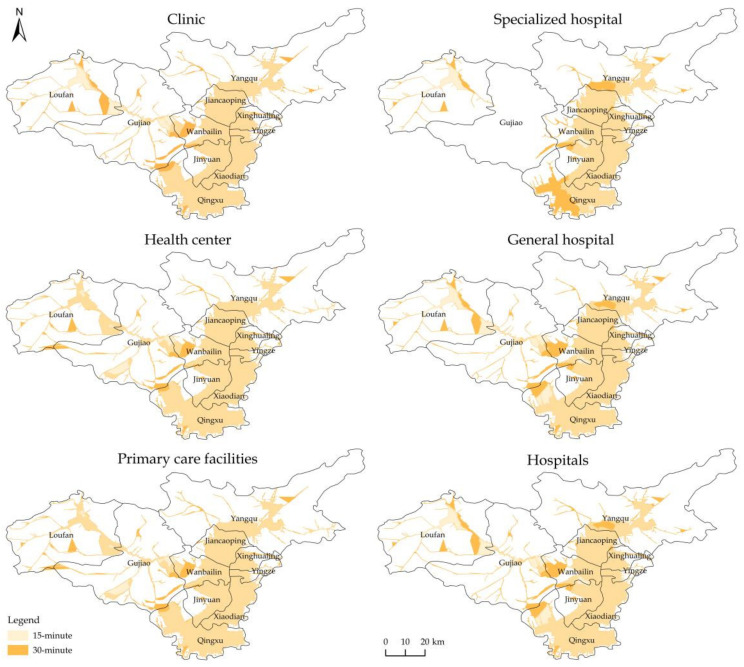
The service area of two-tier and four-type medical and health resources in Taiyuan.

Figure 4 depicts the service areas of all tiers and types of medical and health resources, and Table 5 presents their sizes and ratios. We can conclude that Taiyuan’s overall medical and health resource allocation was relatively low. There was a large discrepancy between the 15 min service area and the requirements of the Healthy Shanxi 2030 Planning Outline. As shown in Table 5, within the 15 min health service area, primary care facilities covered 25.93% of the municipal area, while the hospitals covered 22.63%. Within the 30 min health service area, the coverage of primary care facilities was 30.04%, and that of the hospitals was 26.51%. Although the service area of medical and health resources had been expanding over time, it was still less than one-third of the municipal area, which means that, in most areas, it has not been easy for residents to obtain medical and health services.

The spatial distribution equilibrium of medical and health resources in Taiyuan was poor. The time needed to reach the nearest resource points in the central urban area, in Qingxu, and in areas along high-traffic roads was lower than that in other areas. The central urban area and Qingxu are located on the alluvial plains on both sides of the Fenhe River, with flat terrain and a dense transportation network, so most residents can reach the nearest resource points within 30 min and conveniently obtain medical and health services. However, Loufan, Yangqu, and Gujiao are mountainous, with poor traffic accessibility and with fewer and unevenly distributed medical and health resources. Thus, only the banded area in northeastern Loufan, central and southern Yangqu, central Gujiao, and the areas along high-traffic roads were covered by the 30 min service area, and residents in other areas have difficulty reaching healthcare facilities.

The spatial distribution equilibrium of primary care facilities, such as health centers, was superior to that of hospitals. Health centers were the most widely distributed among the four types of health resources. Social welfare institutions are established by counties or townships. The nonprofit attribute determines their balanced spatial distribution and wide service range. Being the mainstay and foundation of a health service system providing basic health services, lower-level medical and health institutions were abundant, widely distributed, and had a wider service area.

### 4.3. Influencing Factors and Mechanism

#### 4.3.1. Influencing Factors

The Geodetector results of influencing factors of the two-tier medical and health resource spatial distribution in Taiyuan are shown below (Table 6). The comprehensive influence of the economy and the terrain on the spatial distribution of medical and health resources was relatively high, and the influence of each factor was above 0.7. There was a large difference in the influence of various factors in the population system (0.345 < *q* < 0.979). Only the population density (X8) and the population urbanization rate (X9) were above 0.7. In the finance system, fiscal revenue decentralization (X12) and the financial self-sufficiency rate (X14) had a relatively high impact on resource distribution; both were above 0.6. In the medical insurance system, the number of participants in basic medical insurance for urban and rural residents (X15) had a strong influence on the resource spatial distribution (*q* > 0.8), while the participation rate of basic medical insurance for urban and rural residents (X16) had a low influence (*q* < 0.3).

The relief degree of the land surface (X17), the number of participants in basic medical insurance for urban and rural residents (X15), the per capita GDP (X3), the per capita urban disposable income (X5), and the population urbanization rate (X9) were the main factors influencing the spatial distribution of primary care facilities (*q* > 0.9). The main factors affecting the spatial distribution of hospitals (*q* > 0.9) were the population urbanization rate (X9), the proportion of non-agricultural industries (X4), and the financial self-sufficiency rate (X14). The population urbanization rate played a leading role in resource distribution at both tiers. The fiscal expenditure decentralization (X13), the natural population growth rate (X7), the registered population (X6), and the educational level (X11) had a significantly higher influence on the distribution of primary care facilities than that of hospitals, and the q difference was higher than 0.2.

#### 4.3.2. Mechanism

Economic development is the internal motivation for the balanced distribution of medical and health resources. Economically developed areas have a solid development foundation and a good development trend and are more likely to attract medical and health resources, resulting in a spatial agglomeration effect. However, it also leads to the loss of resources in underdeveloped areas, further widening spatial distribution differences. In high-income areas, residents are less apprehensive about medical treatment expenses, which also attracts private medical institutions to increase resource allocation.

A disadvantageous terrain is a natural obstacle to a balanced distribution of medical and health resources. A natural geographical environment is a prerequisite for human production and life. The harsh terrain in mountainous cities increases the cost and difficulty of medical institutions construction and reduces their service radius range, resulting in their uneven spatial distribution. As the service radius of primary care facilities is smaller than that of hospitals, more resources are allocated to meet the needs of the full coverage of medical and health services. However, the high investment and low benefit of resource allocation in mountainous areas exacerbate the imbalance between the supply and demand of primary care facilities, so the influence of terrain factors on the primary care facilities is greater than that of hospitals.

Population is an important consideration for the rational distribution of medical and health resources. Being the market and object of medical and health services, the resident is important for the distribution and survival of medical and health resources. On the one hand, the higher the population density, the greater the demand for medical and health resources, and the more hospitals are distributed. On the other hand, in Loufan, Yangqu, Gujiao, and other remote mountainous areas, the residents are scattered; therefore, by simply considering population density, an uneven resource distribution occurs.

Regional policy plays an important role in regulating and controlling the balanced distribution of medical and health resources. Local finance shows the government’s attention and support of healthcare. Fundamental and guaranteed primary institutions are more affected by economic development and financial support, reflecting the complementary role of policies in meeting livelihood needs in economically underdeveloped regions. Medical insurance is the social welfare of a balanced distribution of medical and health resources. Establishing and improving a medical security system is an important basis for promoting fairness in medical expenses and an effective means of promoting equitable resource allocation. Affected by a higher reimbursement ratio and a lower pay line, the responsivity of primary care facilities to the number of insured people and the insurance rate is greater than that of hospitals.

## 5. Discussion

We found that there were regional differences in the spatial allocation of medical and health resources in Taiyuan, with higher levels of resource allocation in central urban areas and areas along the high-traffic roads, while primary care facilities were poorly distributed. Correspondingly, more than half of the interviewees needed more than 15 min to reach the nearest medical and health institution. Among them, Interviewees 8 and 10, who were aged and needed long-term medication, said that access to medical and health services was inconvenient, and there was an urgent need for home doctor services or additional health centers. This was similar to the findings of Zhifei Ma and Xuchuan Sun [11,32] who showed that medical and health resources were clustered in large cities and central areas. Therefore, for the implementation of basic public health services, it is necessary to strengthen management and redeployment, optimize the spatial distribution of medical and health resources, increase resource supply in surrounding counties, such as Yangqu, Loufan, Gujiao, and Qingxu, and improve the construction of basic medical services in a 15 min radius. In addition, the findings of this paper showed that the quantity and the HRDI of primary care facilities were significantly higher than those of hospitals, and the spatial distribution equilibrium was also superior to that of hospitals, contrary to the findings of Junhao Wang and Xueqian Song et al., who found that primary institutions suffered from insufficient allocation [17,18]. Perhaps due to the typical primacy distribution of the urban scale in Shanxi, human, material, and financial resources within medical and health resources tend to be concentrated at hospitals, resulting in a low level of equilibrium with little disparity in human, material, and financial resources within primary medical and health resources. Most interviewees understood the tiered healthcare concept and were able to access different tiers of medical and health institutions according to the severity of their disease and the difficulty of treatment. Therefore, it is necessary to continuously improve the tiered healthcare system, to establish a scientific and effective medical order featuring primary treatments at primary institutions, two-way referral, tier linkage, and a separation of emergency and slow treatment, and to meet the different medical needs of residents.

The spatial distribution of medical and health resources is the result of a combination of factors, and the level of economic development is the most important factor. The results of the analysis of influencing factors in this paper are similar to those of others [18,20,21,22,23,24]. It is worth noting that this paper expands on the existing literature by adding the factor of the relief degree of land surface. The study showed that the relief degree of land surface had a significant influence on two-tier resource allocation. The main reason is that Taiyuan, as a mountainous city, has a large relief and elevation difference, and the mountainous and hilly terrain covers about four-fifths of the total area. The terrain conditions cannot be ignored when analyzing the factors influencing the spatial distribution of medical and health resources in Taiyuan. In addition, the interview results revealed that, in addition to the high expense, the relatively long distance, inaccessibility, and limited level of treatment were also the main reasons for the difficulty in accessing medical and health services. This was strongly related to the low level of local medical and health resource allocation and the predominantly mountainous topography, which is consistent with the analysis of the Geodetector results. Therefore, Taiyuan should focus on increasing resource supply in remote mountainous areas and rationally allocating resources considering the terrain and population. Terrain disadvantages can be compensated for through transport improvement, financial subsidies, talent support, information sharing, and so on. Efforts to promote economic development and industrial optimization, to increase household income, and to improve the medical security system must continue being made.

The widespread outbreak of COVID-19 increased the demand for medical and health resources, and the resource gap has expanded further. Most interviewees stated that medical treatment was more difficult after the COVID-19 outbreak. In order to ensure the needs for epidemic prevention, control, and isolation, as well as the routine diagnosis and treatment of chronic and acute diseases, epidemic prevention and control should not rely solely on conventional medical resources. Medical and health resources should be prepared for the emergency prevention and control of public infectious diseases. Large public places, such as stadiums and exhibition centers, should be selected for the reconstruction of cabin hospitals, so that emergency medical rescue tasks can be undertaken, and basic medical services can be provided.

## 6. Conclusions

(1) Taiyuan suffers from a poor allocation of medical and health resources, and the spatial distribution is uneven, as only residents in central urban areas and areas along the high-traffic roads had convenient access to medical and health services. The spatial distribution balance of primary care facilities is superior to that of hospitals. The economy and terrain have a high impact on the spatial distribution of medical and health resources, and the responsivity of primary care facilities is greater than that of hospitals. The spatial distribution of medical and health resources is the result of a combination of factors. For a balanced distribution of medical and health resources, economic development is the internal motivation, a disadvantageous terrain is a natural obstacle, the population is an important condition, and regional policies play an important regulatory role.
(2) Spatial quantity analysis, spatial distribution analysis, and Geodetector analysis can indicate the spatial allocation equilibrium characteristics of two-tier medical and health resources in Taiyuan, identify main factors affecting their allocation, and improve the scientific basis for an even allocation of medical and health resources. This paper can help in promoting the improvement of the tiered healthcare system in Taiyuan, improving the resource allocation equilibrium, and satisfying medical treatment demand at different levels. This paper has reference value in terms of carrying out relevant research in other regions.
(3) There are limitations in this study. For example, due to the lack of medical and health statistics at the smaller regional scale in Taiyuan, the research scale of this study was at the district and county level. More detailed analyses, such as townships or census areas, were lacking, and only the quantity and spatial distribution characteristics of medical and health resources were assessed. Due to time constraints and China’s dynamic zero-COVID policy, interviews were difficult. Only one typical village was selected for interviews, and the number of interviewees was limited. Therefore, in the future, researchers can collect complete data, refine the research scale, and investigate the differences in the quantity and quality of tiered resources. In terms of the time and policy conditions permitted, in order to gain a more realistic and extensive understanding of the problems in medical and health resource allocation and provide a scientific reference for the optimization of medical and health resources in Taiyuan, the scope of interviews can be expanded, and the number of interviewees can be increased.

## Figures and Tables

**Table 1:** Tier and type of POI data of medical and health resources in Taiyuan.

Tier	Type	Number	POI
Primary care facilities	Clinic	1287	clinic
	Health center	331	health center
Hospitals	Specialized hospital	104	brain hospital; chest hospital; ENT hospital; eye hospital; Grade III-A specialized hospital; gynecological hospital; infectious diseases hospital; orthopedics hospital; psychiatric hospital; specialized hospital; stomatological hospital; tumor hospital
	General hospital	157	general hospital; Grade III-A general hospital

**Table 2:** Index system of influencing factors on the spatial distribution equilibrium of medical and health resources.

Detection System	Detection Factor
Economy	X1 GDP (CNY 10,000)
	X2 per capita GDP (CNY)
	X3 GDP per km^2^ (CNY 10,000)
	X4 proportion of non-agricultural industries (%)
	X5 per capita urban disposable income (CNY)
Population	X6 registered population (person)
	X7 natural population growth rate (‰)
	X8 population density (person per km^2^)
	X9 population urbanization rate (%)
	X10 population mortality rate (‰)
	X11 educational level (%, proportion of primary and secondary school students in the total population)
Finance	X12 fiscal revenue decentralization (%, proportion of district or county financial revenue in Taiyuan’s financial revenue)
	X13 fiscal expenditure decentralization (%, proportion of district or county financial expenditure in Taiyuan’s financial expenditure)
	X14 financial self-sufficiency rate (%, proportion of financial revenue to financial expenditure)
Medical insurance	X15 number of participants in basic medical insurance for urban and rural residents (person)
	X16 participation rate of basic medical insurance for urban and rural residents (%)
Terrain	X17 relief degree of land surface (meter)

**Table 3:** Speed assignment of roads.

Road	Expressway	National Highway	Provincial Highway	County Highway	Township Road	Urban Road I	Urban Road II	Urban Road III	Urban Road IV
Speed (km/h)	100	80	60	40	30	80	60	40	30

**Table 4:** Spatial quantity distribution of two-tier medical and health resources in Taiyuan.

Region	The per Capita Index			The Area Mean Index			HRDI		
	Whole	Primary Care Facilities	Hospitals	Whole	Primary Care Facilities	Hospitals	Whole	Primary Care Facilities	Hospitals
Taiyuan	0.490	0.422	0.068	0.269	0.232	0.037	0.363	0.313	0.050
Xiaodian	0.850	0.735	0.115	2.000	1.729	0.271	1.304	1.127	0.177
Wanbailin	0.537	0.480	0.057	1.043	0.931	0.111	0.748	0.668	0.080
Xinghualing	0.379	0.326	0.053	1.382	1.188	0.194	0.724	0.622	0.102
Yingze	0.409	0.312	0.097	1.915	1.462	0.453	0.885	0.675	0.209
Jiancaoping	0.459	0.402	0.057	0.537	0.470	0.067	0.497	0.435	0.062
Jinyuan	0.543	0.473	0.070	0.406	0.354	0.052	0.470	0.409	0.060
Qingxu	0.312	0.286	0.027	0.174	0.159	0.015	0.233	0.213	0.020
Gujiao	0.289	0.242	0.047	0.039	0.033	0.006	0.106	0.089	0.017
Yangqu	0.255	0.222	0.033	0.019	0.017	0.002 ^①^	0.069	0.061	0.009
Loufan	0.279	0.255	0.024	0.027	0.025	0.002 ^②^	0.087	0.080	0.007

Note: The actual data of Equations ① and ② are 0.00243 and 0.00235, which were set to three decimal digits for the uniformity and simplicity of the table data.

**Table 5:** The service area sizes and their ratios of two-tier and four-type medical and health resources in Taiyuan.

Tier	Type	15 min Service Area Size/km^2^	15 min Service Area Size Ratio/%	30 min Service Area Size/km^2^	30 min Service Area Size Ratio/%
Primary care facilities	Clinic	1665.15	23.83	1924.94	27.55
	Health center	1807.10	25.86	2084.08	29.82
	Subtotal	1811.77	25.93	2099.24	30.04
Hospitals	Specialized hospital	1252.21	17.92	1594.52	22.82
	General hospital	1580.52	22.62	1841.48	26.35
	Subtotal	1581.19	22.63	1852.52	26.51

**Table 6:** Geodetector results of influencing factors of two-tier medical and health resource spatial distribution in Taiyuan.

Detection System	Detection Factor	Primary Care Facilities			Hospitals		
		*Q*	*q* Ranking	*p*	*q*	*q* Ranking	*p*
Economy	X1	0.852	8	0.000	0.740	9	0.000
	X2	0.886	6	0.000	0.857	5	0.000
	X3	0.910	3	0.000	0.776	7	0.000
	X4	0.854	7	0.000	0.950	2	0.000
	X5	0.903	4	0.000	0.715	10	0.000
Population	X6	0.838	9	0.000	0.574	12	0.000
	X7	0.694	13	0.000	0.404	15	0.000
	X8	0.831	10	0.000	0.873	4	0.000
	X9	0.902	5	0.000	0.979	1	0.000
	X10	0.345	16	0.000	0.409	14	0.000
	X11	0.737	12	0.000	0.528	13	0.000
Finance	X12	0.677	14	0.000	0.636	11	0.000
	X13	0.674	15	0.000	0.369	16	0.000
	X14	0.790	11	0.000	0.922	3	0.000
Medical insurance	X15	0.917	2	0.000	0.845	6	0.000
	X16	0.267	17	0.000	0.196	17	0.000
Terrain	X17	0.930	1	0.000	0.760	8	0.000

## Data Availability

The original database used is publicly available at AMAP https://restapi.amap.com and https://ditu.amap.com (accessed on 16 April 2021), https://www.webmap.cn/main.do?method=index (accessed on 19 November 2021), Relief Degree of Land Surface Dataset of China (1 km) and Taiyuan Statistical Yearbook in 2020.
